# A Bayesian bird's eye view of ‘Replications of important results in social psychology’

**DOI:** 10.1098/rsos.160426

**Published:** 2017-01-18

**Authors:** Maarten Marsman, Felix D. Schönbrodt, Richard D. Morey, Yuling Yao, Andrew Gelman, Eric-Jan Wagenmakers

**Affiliations:** 1 Department of Psychology, University of Amsterdam, Amsterdam, The Netherlands; 2 Department of Psychology, Ludwig-Maximilians-Universität München, Munchen, Germany; 3 School of Psychology, Cardiff University, Cardiff, UK; 4 Department of Statistics, Columbia University, New York, NY, USA

**Keywords:** preregistration, evidence, reproducibility, credible interval, Bayes factor

## Abstract

We applied three Bayesian methods to reanalyse the preregistered contributions to the *Social Psychology* special issue ‘Replications of Important Results in Social Psychology’ (Nosek & Lakens. 2014 Registered reports: a method to increase the credibility of published results. *Soc. Psychol.* **45**, 137–141. (doi:10.1027/1864-9335/a000192)). First, individual-experiment Bayesian parameter estimation revealed that for directed effect size measures, only three out of 44 central 95% credible intervals did not overlap with zero and fell in the expected direction. For undirected effect size measures, only four out of 59 credible intervals contained values greater than 0.10 (10% of variance explained) and only 19 intervals contained values larger than 0.05. Second, a Bayesian random-effects meta-analysis for all 38 *t*-tests showed that only one out of the 38 hierarchically estimated credible intervals did not overlap with zero and fell in the expected direction. Third, a Bayes factor hypothesis test was used to quantify the evidence for the null hypothesis against a default one-sided alternative. Only seven out of 60 Bayes factors indicated non-anecdotal support in favour of the alternative hypothesis (${\mathrm{BF}}_{10}>3$), whereas 51 Bayes factors indicated at least some support for the null hypothesis. We hope that future analyses of replication success will embrace a more inclusive statistical approach by adopting a wider range of complementary techniques.

## 1. Introduction

Skillfully conducted replication studies can greatly influence researchers' confidence in the presence, impact and general nature of a hypothesized effect. But how should replication studies be conducted? A recent special issue in *Social Psychology* showed by example how informative replication studies can be designed [1, 2]. In the special issue, the trinity of replication guidelines was to collaborate with original authors, to use preregistration and to conduct high-powered studies (see also [3–5]). Therefore, the replication studies in the special issue were conducted under relatively ideal circumstances.

Although the special issue has attracted widespread attention, the data have not yet been analysed as a whole, across all replication attempts. In addition, the individual replication attempts were analysed solely with classical statistics (i.e. *p*-values and confidence intervals). Classical methods, however, are unable to quantify evidence; in particular, classical methods cannot distinguish between the absence of evidence (i.e. the data are uninformative) or the evidence of absence (i.e. the data support a point null hypothesis $H_{0}$). Hence it is possible that even a high-powered replication with a non-significant *p*-value can be evidentially uninformative for the question of interest [6, 7].

Consequently, our goals are twofold. Our primary goal is to provide a Bayesian bird's eye perspective on the results from the *Social Psychology* special issue [1]. Specifically, we reanalyse the results from the individual contributions to the special issue using three Bayesian methods. First, an individual-study parameter estimation approach yields posterior distributions of effect size for each study considered in isolation; these posterior distributions quantify our uncertainty about the key quantity of interest—the narrower the posterior distribution for effect size, the more certain we can be about its value. Second, a hierarchical parameter estimation approach also yields posterior distributions for effect size, but it does not consider the studies in isolation; instead, the hierarchical approach implements group-level constraints and conceptualizes each individual study's effect size as a draw from a group-level normal distribution whose variance reflects the heterogeneity between studies. Third, a hypothesis testing approach quantifies evidence for the point null hypotheses versus a specific one-sided alternative hypothesis. This cannot be accomplished by Neyman–Pearson's style hypothesis testing, whose explicit goal it is to control error rate in repeated use. However, as emphasized by the editors of the *Social Psychology* special issue, confidence in scientific claims is based on ‘evaluating the evidence’ ([1, p. 139]; for a summary of other reasons to consider a Bayesian analysis, see e.g. [8, 9]).^1^ As explained below, all three Bayesian approaches presented here follow from the same coherent framework in which knowledge about parameters and hypotheses is updated based on predictive success.

Our secondary goal is to highlight the feasibility of analysing data from standard experiments in social psychology using Bayesian tools. Specifically, we analyse the special issue data using JASP ([10], jasp-stats.org), Stan [11–13] and R ([14], especially the BayesFactor package). Armed with these software programs, Bayesian methods can be easily applied to a series of common analyses such as the *t*-test, contingency tables, regression and analysis of variance (ANOVA).

## 2. Brief Bayesian background

At its core, Bayesian inference requires only that the user creates a ‘generative’ statistical model, that is, a model that makes predictions about to-be-observed data. Once a particular dataset is observed, Bayes' rule inverts the generative model and updates the uncertainty about the model parameters in a coherent fashion. The updated model makes new predictions about to-be-observed data, and the predict–observe–update cycle of Bayesian inference can continue indefinitely as the data accumulate [15]. The central aspect of Bayesian inference therefore is prediction: it is predictive performance that drives the coherent update of knowledge. We demonstrate this point by application to two key Bayesian tasks: parameter estimation and hypothesis testing.

### 2.1. Bayesian parameter estimation: the basic concepts

In order to have a model make predictions, its parameters need to be assigned particular values. Often we do not know exactly what these values are—they are the very entities we want to learn about. Consequently, Bayesians assign prior distributions to parameters *θ* in order to reflect the uncertainty about their true value. These prior distributions p(θ) quantify one's knowledge about the unknown parameters *θ* before seeing the data. After seeing the data, the prior distribution p(θ) is updated to a posterior distribution p(θ∣data): the uncertainty about *θ* ‘given’ the data. The updating proceeds by assessing predictive success, as can be seen by writing Bayes' rule as follows [16, 17]:

2.1 $$\underset{\begin{array}{c} \mathrm{posterior}\;\mathrm{knowledge} \end{array}}{\underbrace{p(\theta |\mathrm{data})}}=\underset{\begin{array}{c} \mathrm{prior}\;\mathrm{knowledge} \end{array}}{\underbrace{p(\theta )}}\times \underset{\begin{array}{c} \mathrm{predictive}\\ \mathrm{updating}\;\mathrm{factor} \end{array}}{\underbrace{\frac{p(\mathrm{data}|\theta )}{p(\mathrm{data})}}}.$$

This equation shows that the update from prior to posterior distribution is governed by a predictive updating factor; this factor considers, for each value of *θ*, its predictive success p(data∣θ)—that is, the probabilistic forecast for the observed data according to a specific *θ*. This predictive success for a specific *θ* is then assessed relative to the average predictive success p(data)—the probabilistic forecast for the observed data across all values of *θ*. Hence, the Bayesian updating process is guided by predictive success: parameters that predict well receive a boost in plausibility, whereas parameters that predict poorly suffer a decline [15–17].

Below we apply the Bayesian parameter estimation framework to the studies published in the *Social Psychology* special issue. For this purpose it is convenient to summarize the posterior distribution by its location (i.e. the posterior median) and spread (i.e. a 95% central credible interval). In the Bayesian framework, the interpretation of these summary values is intuitive and direct: given the data and the statistical model—which includes the specification of the prior distribution as well as the likelihood—we can be 50% confident that the true value is higher or lower than the median, and we can be 95% confident that the true value lies in the interval [18, 19].

The prior distributions have been assigned by default, depending on general desiderata inherent to the Jeffreys–Zellner–Siow framework [20–26]. Note that the posterior distribution is a compromise between the prior and the data, and therefore—as long as the data are sufficiently informative—the posterior distribution will be relatively robust to changes in the specification of the prior distribution.

Below we report posterior distributions for both directed and undirected effect size measures. The directed effect size measures include *δ* for *t*-tests and *ρ* for correlation tests, and the undirected effect size measures include Cramér's $\phi^{2}$ for contingency tables [27, p. 282], and $\rho^{2}$ for *t*-tests, correlation tests and ANOVAs (e.g. [28]). For the *t*-test and ANOVA, $\rho^{2}$ has the same interpretation as $\omega^{2}$ and $\eta^{2}$—the proportion of variance explained by the experimental design. When more than one experimental factor is used, we report the squared semi-partial correlation to quantify the unique contribution of the primary experimental factor of interest.

We produced estimates for the directed effect sizes *δ* and *ρ* using JASP and produced estimates for the undirected effect sizes $\phi^{2}$ and $\rho^{2}$ using the BayesFactor package [14] in R. We have made the JASP files, data and R-code available at https://osf.io/bqwzd/.

### 2.2. Bayesian parameter estimation: hierarchical models

The Bayesian parameter estimation approach detailed above can be gracefully extended to a hierarchical model, in which inference for individual studies is informed and constrained by a single overarching distribution: the group-level model [8, 29, 30]. Hierarchical analyses such as the one applied in our reanalysis have three main benefits [31]. First, the individual-study results contribute to the estimation of group-level parameters that describe both the heterogeneity between studies and the group mean effect. Second, the group-level structure shrinks individual results that are uncertain and relatively extreme towards the group mean (e.g. [32]). Third, the uncertainty about individual studies is generally reduced when information is borrowed from other, statistically similar studies, which is expressed in narrower posterior credible intervals. In our reanalysis below, we use this hierarchical approach for the available 38 *t*-tests reported in the special issue, as imposing a hierarchical structure on the effect sizes *δ* is simple and intuitive.

For the hierarchical analysis on effect size from the 38 *t*-test studies, we use the model formulation from Rouder *et al.* [23]. For the one sample *t*-test, the mean in study *s* is parametrized as $\sigma_{s}\delta_{s}$, where $\delta_{s}$ is the standardized effect size. For an independent *t*-test, the mean for group 1 is $\mu_{s}+1/2\sigma_{s}\delta_{s}$ and the mean for group 2 is $\mu_{s}-1/2\sigma_{s}\delta_{s}$, and $\sigma_{s}^{2}$ indicates the common variance. For the one sample *t*-test and the independent *t*-test, the key parameter of interest is the standardized effect size $\delta_{s}$. In the hierarchical model, it is assumed that the effect sizes come from a single overarching distribution, that is, the group-level model. Note that many experiments from the special issue concern phenomena that are conceptually unrelated; consequently, the group-level distribution describes the location and heterogeneity of ‘important results in social psychology’ that were deemed suitable for preregistered replication. As will be evident below, the heterogeneity in effect sizes is estimated to be relatively small, and this results in a substantial shrinkage effect.

One reviewer objected to the use of a hierarchical model for effects that are conceptually unrelated. This is a venerable issue (e.g. see [32] for an extended discussion) and we offer the following motivation. First, the hierarchical model contains a parameter that measures the heterogeneity across studies. In our application, the studies turn out to be highly homogeneous. This does not show that the studies are conceptually related, but it does show that their effect sizes are highly similar, and this is all that is required for an application of the model. In other words, the model assumes only that the effect sizes across studies are statistically similar, and does not speak to the degree of conceptual similarity. Second, the extent to which effects are conceptually related is not an all-or-none matter. One may always argue that the individual case is unique and, at some level, conceptually unrelated to the other cases. However, all studies considered here come from social psychology and have been submitted to the same special issue. We believe this commonality warrants the application of the hierarchical model.

A limitation of our Bayesian random-effects meta-analysis is that it assumes that the 38 effect sizes *δ* are independent realizations from a single overarching distribution. This is of course not entirely the case, as several *t*-tests were used to test the same hypothesis (e.g. the analyses by IJzerman *et al.* [33]), or were used to test an effect on several measures within the same experiment (e.g. the analyses by Johnson *et al.* [34]). However, a dataset of 38 *t*-tests will only admit a model of limited complexity, and we believe that our model achieves the right balance in the inevitable trade-off between bias and variance (e.g. [35]). Our assessment is bolstered by the fact that for the dataset under consideration, there are relatively few ‘duplicates’, and there is relatively little heterogeneity across effect sizes; consequently, there is almost no information available to support the inclusion of additional topic-specific parameters.

We assume here that the standardized effect sizes $\delta_{s}$ follow a normal distribution with an unknown group mean *θ* and variance (i.e. study heterogeneity) $\tau^{2}$. To complete the Bayesian hierarchical model, we have used standard non-informative priors on the individual-study means $\mu_{s}$, individual-study variances $\sigma_{s}$ and the group-level variance $\tau^{2}$ (i.e. $p(\mu_{s},\sigma_{s})\propto \sigma_{s}^{-2}$ and $p(\tau )\propto \tau^{-2}$), and have used a Cauchy$\left(0,1/\sqrt{2}\right)$ prior on the group-level mean *θ* that is in line with the prior on effect sizes *δ* in the individual analyses. The analysis was performed using the R-package rstan [11–13], with the data and R-code available at https://osf.io/bqwzd/.

### 2.3. Bayesian hypothesis testing: Bayes factors

The predictive framework that governs the coherent plausibility updates for parameters carries over seamlessly to plausibility updates for entire models or hypotheses. To see this, we again use Bayes' rule and obtain

2.2 $$\underset{\begin{array}{c} \mathrm{posterior}\;\mathrm{knowledge}\\ \mathrm{about}\;\mathrm{hypotheses} \end{array}}{\underbrace{\frac{p(H_{1}|\mathrm{data})}{p(H_{0}|\mathrm{data})}}}=\underset{\begin{array}{c} \mathrm{prior}\;\mathrm{knowledge}\\ \mathrm{about}\;\mathrm{hypotheses} \end{array}}{\underbrace{\frac{p(H_{1})}{p(H_{0})}}}\times \underset{\begin{array}{c} \mathrm{predictive}\\ \mathrm{updating}\;\mathrm{factor} \end{array}}{\underbrace{\frac{p(\mathrm{data}|H_{1})}{p(\mathrm{data}|H_{0})}}}.$$

As was the case for parameter estimation, the update from prior plausibility to posterior plausibility is governed by predictive success: the hypothesis that predicts the observed data better than the competitor hypothesis receives a boost in plausibility (e.g. [15, 36]).

Note that the framework is inherently relative: what matters is which of the two hypotheses does best, not whether a specific hypothesis does well in an absolute sense. Also note that the predictive focus means that the results do not depend on one of the statistical models being ‘true’ in some abstract sense. This latter point is particularly relevant in the context of a point null hypothesis, which many have argued is an unlikely proposition on *a priori* grounds [37, 38]. For the interpretation of the Bayes factor, however, it does not matter whether the point null hypothesis (or the alternative hypothesis against which it is pitted) is unlikely to be true in an absolute sense; indeed, all models are abstraction of reality and are therefore likely to be ‘wrong’. However, in a predictive sense the point null hypothesis can be a good approximation for an effect that is so small that it cannot be detected reliably. As remarked by Andrew Gelman, ‘when effect size is tiny and measurement error is huge, you're essentially trying to use a bathroom scale to weigh a feather—and the feather is resting loosely in the pouch of a kangaroo that is vigorously jumping up and down.’^2^ In such situations, the point null hypothesis will predictively outperform the alternative hypothesis.

Equation (2.2) quantifies the adage ‘extraordinary claims require extraordinary evidence’; in Bayesian terms, this translates to the statement ‘An implausible hypothesis requires substantial predictive success’. The quantification of prior implausibility of a hypothesis is subjective and may depend on many unknowns. We therefore follow standard Bayesian practice and quantify only the predictive updating factor, that is, the degree to which the data change the relative plausibility of the hypotheses under consideration.

Thus, for the studies from the special issue in *Social Psychology* we report the predictive updating factor

$${\mathrm{BF}}_{10}=\frac{p(\mathrm{data}|H_{1})}{p(\mathrm{data}|H_{0})},$$

which is commonly known as the Bayes factor^3^ [21, 43]. The result ${\mathrm{BF}}_{10}=2$ indicates that the observed data are twice as likely under $H_{1}$ than under $H_{0}$; the result ${\mathrm{BF}}_{10}=0.5$ indicates the exact opposite. Harold Jeffreys proposed a set of descriptive categories of evidential impact, and proposed that Bayes factors in between 3 and 1/3 are ‘not worth more than a bare mention’ [21, appendix B]. Although the interpretation of evidence does not require arbitrary category labels, they do facilitate a concise summary. As in the case of parameter estimation, we use the default Jeffreys–Zellner–Siow priors, with the exception that the priors are specified to be one-sided, respecting the fact that in replication research the hypothesis of interest is directional. Other prior choices are possible and, to the extent that they ask a slightly different question, they might lead to a slightly different answer (e.g. [7, 44–47]). The default choices in the present work are built into the BayesFactor package for R [14] and the companion software JASP [10].

### 2.4. Single study example

In order to explain our reanalysis approach in more concrete terms, consider the following example. Shackelford *et al.* [48] reported that men were more distressed by sexual infidelity than women, an effect that IJzerman *et al.* [33] sought to replicate in several studies reported in the *Social Psychology* special issue. Their first replication study featured 18 men and 69 women, and the results showed that compared to the women, the men had significantly lower sexual dilemma scores (SDS; $t_{85}=4.178$, p<0.001, d=1.106), where lower SDS-scores indicate higher distress.^4^ Below we report the Bayesian results for single-study parameter estimation and for hypothesis testing.

**Figure 1.**
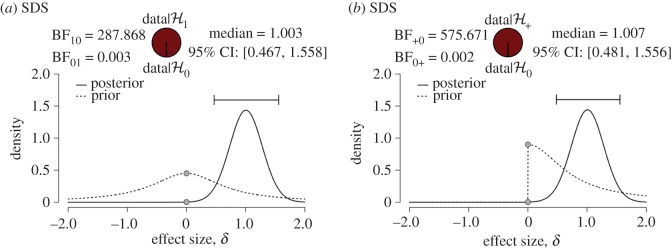
JASP output for the *t*-test example featuring the first replication study reported in IJzerman *et al.* [33]. Both panels show the prior and posterior distributions for effect size, the posterior median, the central 95% credible interval and the Bayes factor. (*a*) The results for the unrestricted hypothesis $H_{1}$ and (*b*) the results from the directional hypothesis $H_{+}$.

Concerning Bayesian parameter estimation, the left panel of figure 1 shows the prior distribution (dotted line) and the posterior distribution (solid line) for the effect size *δ* based on the data from the first replication study reported in IJzerman *et al.* [33]. The data have caused the prior distribution to undergo a substantial update, indicating that the data were highly informative. The posterior median is 1.00 and the central 95% credible interval equals [0.47,1.56]. In addition to a directed effect size *δ* we also report the undirected effect size $\rho^{2}$, which quantifies the proportion of the variance of sexual dilemma scores that can be explained by gender differences. In this example, the posterior median for $\rho^{2}$ is 0.14 (i.e. 14% variance explained) and the central 95% credible interval equals [0.03,0.29]. These are the posterior summary measures that we report below for all available studies in the *Social Psychology* special issue, and later we also report the results from a hierarchical analysis that considers multiple studies simultaneously.

Concerning Bayesian hypothesis testing, the left panel in figure 1 shows the Bayes factor ${\mathrm{BF}}_{10}$. This Bayes factor contrasts the predictive success of the alternative hypothesis $H_{1}$ (i.e. SDS-scores differ between men and women) with that of the null hypothesis $H_{0}$ (SDS-scores do not differ between men and women). The result, ${\mathrm{BF}}_{10}\approx 288$, indicates that the observed data are almost 288 times more likely to occur under $H_{1}$ than under $H_{0}$, providing very strong support for the alternative hypothesis.

However, the replication study did not aim to test whether SDS-scores differ between men and women; instead, its aim was to test a directional hypothesis, namely that men have lower SDS-scores than women (i.e. men are more distressed than women about sexual infidelity, not less). We denote the directional hypothesis as $H_{+}$. The directional nature of the hypothesis can be incorporated into the analysis by folding the prior distribution such that it only has mass for effect sizes in the predicted direction [49]. The right panel of figure 1 shows the prior and posterior distributions produced by this directional hypothesis. The associated Bayes factor ${\mathrm{BF}}_{+0}$ contrasts the directional alternative hypothesis $H_{+}$ against the null hypothesis $H_{0}$. The result, ${\mathrm{BF}}_{+0}\approx 576$, indicates that the observed data are about 576 times more likely to occur under $H_{+}$ than under $H_{0}$, again providing very strong support for the alternative directional hypothesis. Note that in this specific case the evidence is almost twice as strong for the directional hypothesis $H_{+}$ (figure 1*b*) than it is for the unrestricted hypothesis $H_{1}$ (figure 1*a*), despite the fact that the posterior distributions for effect size are virtually identical. The reason is that the directional hypothesis makes predictions that are more daring than those of the unrestricted hypothesis; when the effect goes in the expected direction, the daring predictions are validated and the associated gain in plausibility is, therefore, higher. This provides an example of how Bayes factors quantify the idea that risky scientific predictions ought to be rewarded more than vague scientific predictions (e.g. [50]). The directional Bayes factor ${\mathrm{BF}}_{+0}$ is the measure of evidence that we report below for all available studies in the *Social Psychology* special issue.

We have demonstrated that the Bayes factor for the null hypothesis against an alternative hypothesis depends partly on the predictions for effect size under that alternative hypothesis. These predictions are a direct consequence of the prior distribution that is assigned to effect size; for instance, a two-sided prior yielded ${\mathrm{BF}}_{10}\approx 288$, whereas the one-sided prior gave ${\mathrm{BF}}_{+0}\approx 576$. As mentioned above, for the analysis of the studies from the special issue we use default specifications designed to meet general desiderata (e.g. [20, 51]). However, it is possible to entertain a range of alternative prior specifications and examine the robustness of the conclusions. The standard method to conduct such a robustness check or sensitivity analysis is to vary the width of the prior distribution and consider the resultant change in the Bayes factor.

**Figure 2.**
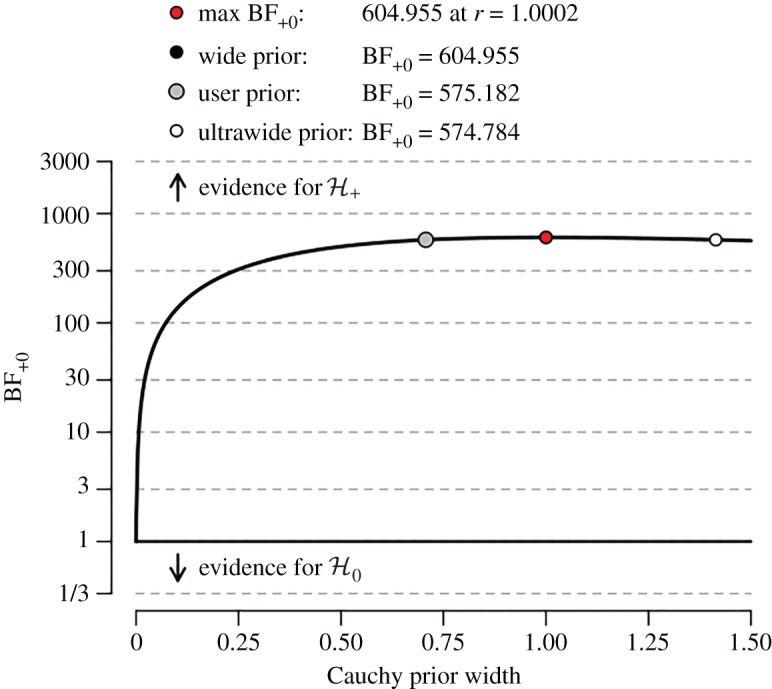
JASP robustness analysis of the *t*-test example featuring the first replication study reported in IJzerman *et al.* [33]. The evidence in favour of the directional hypothesis $H_{+}$ varies as a function of the width of the prior distribution for effect size (i.e. ‘Cauchy prior width’, on the *x*-axis). When the width equals zero, $H_{0}$ and $H_{+}$ are identical and the Bayes factor is 1 regardless of the data. Here the evidence in favour of $H_{+}$ is compelling, with Bayes factors exceeding 100 for all but the most narrow priors. See text for details.

We discuss the pros and cons of such a robustness check by applying it to the IJzerman experiment. Figure 2 shows the associated output from the JASP ‘SumStats’ module.^5^ As dictated by the directionality of the hypothesis under scrutiny, the prior distribution on effect size only assigns mass to positive values. What varies on the *x*-axis is the width of the prior distribution under $H_{+}$. Figure 2 indicates that—for all but the smallest values of the prior width—there is compelling evidence for $H_{+}$ over $H_{0}$ in the sense that the observed data are hundreds of times more likely under $H_{+}$ than under $H_{0}$. The red dot indicates that the Bayes factor is highest for a width of r=1.0002, where it equals about 605; coincidentally, this post hoc prior width is almost exactly the same as the value of the ‘wide prior’ (i.e. r=1) which was originally proposed as a useful default by Jeffreys [21]; consequently, the red dot obscures the black dot. The grey dot indicates the ‘user prior’, which is the modern-day default of $r=2^{-0.5}\approx 0.707$ [14].

Despite the fact that the evidence is compelling across a wide range of prior widths, figure 2 also shows that for small values of the width, the evidence is rather weak. As the width approaches zero, the Bayes factor approaches 1, and *in extremis*, when r=0, the Bayes factor equals 1 irrespective of the data. This occurs because when r=0, the alternative hypothesis $H_{+}$ has morphed into the null hypothesis $H_{0}$, and two models that make identical predictions can never be discriminated based on empirical data. This is an important realization because some researchers may feel the need to reduce the default width in order to accommodate a more modest expectation about effect size. Although a careful subjective specification of prior distributions is generally advantageous (particularly when it occurs in advance of seeing the data), figure 2 indicates that there is a danger when such specification is done solely with respect to the prior width [9]; the default priors are centred on zero and reducing the width will make $H_{+}$ increasingly similar to $H_{0}$. We surmise that in situations where the researcher is unhappy about the default prior width, the researcher is also unhappy about the default prior location—note that under the default specification, the most likely value of effect size under the alternative hypothesis is zero, and absolute values are always more likely the closer they are to zero. Ideally then, a subjective specification ought to take into account both the width and the location of the prior distribution for effect size under the alternative hypothesis.

The current functionality of the JASP program offers only a sensitivity analysis with respect to the prior width. Although informative, this procedure is limited and results for effect sizes near zero should be interpreted with considerable care.^6^ For this reason, and in order not to overwhelm the reader, below we report only the results for the default prior setting. However, the JASP analysis files do contain the sensitivity analyses as shown in figure 2 for the data from the study by IJzerman *et al.* [33].

## 3. Results for all studies

Our reanalysis of the results from IJzerman *et al.* [33, Study 1] indicates compelling statistical support for a replication of the original findings [48]: Bayesian parameter estimation showed the posterior distribution for the effect size *δ* to be away from zero, and Bayesian hypothesis testing confirmed that the default one-sided alternative hypothesis made predictions that were superior to those from the null hypothesis.

We now report the results from a Bayesian reanalysis of the main results across the replication studies in the *Social Psychology* special issue^7^ with the exception of the ManyLabs project [54].

The ManyLabs project was excluded for several reasons. First, the ManyLabs project mostly contained replications of benchmark findings outside of social psychology. Second, for every finding under scrutiny the ManyLabs project featured many replication attempts, and this demands a different analysis approach from the one that is appropriate for single replication attempts. Finally, the results from the ManyLabs project do not much benefit from a sophisticated statistical reanalysis: the conclusions are already evident from a plot of effect sizes reported across the many participating laboratories (i.e. [54, fig. 1]). In other words, when a finding has been subjected to multiple replication attempts the data are bound to pass Berkson's interocular traumatic test, when the conclusion hits one straight between the eyes [39].

Most of the Bayesian reanalyses presented below have been produced with JASP [10]. For analyses currently unavailable in JASP, we mostly used the BayesFactor R-package [14]. Specifically, the BayesFactor package was used to produce the $\rho^{2}$ and $\phi^{2}$ effect size estimates, and to produce some of the Bayes factors involving directional hypotheses for ANOVAs and contingency tables. Finally, the hierarchical analysis was programmed in Stan [11–13]. For each study, the entire reanalysis—the dataset, the R code and the JASP files with input options—is made available through the Open Science Framework (https://osf.io/bqwzd/).

### 3.1. Results from Bayesian parameter estimation: individual studies

This section summarizes the results for individual-study parameter estimation of the contributions to the *Social Psychology* special issue. First, we discuss directed effect sizes such as *δ* and *ρ*, which originate from *t*-tests and correlation tests, respectively. Next, we discuss undirected effect sizes such as $\rho^{2}$ that originate from *t*-tests, ANOVAs, correlation test and contingency tables.

#### 3.1.1. Directed effect sizes.

**Figure 3.**
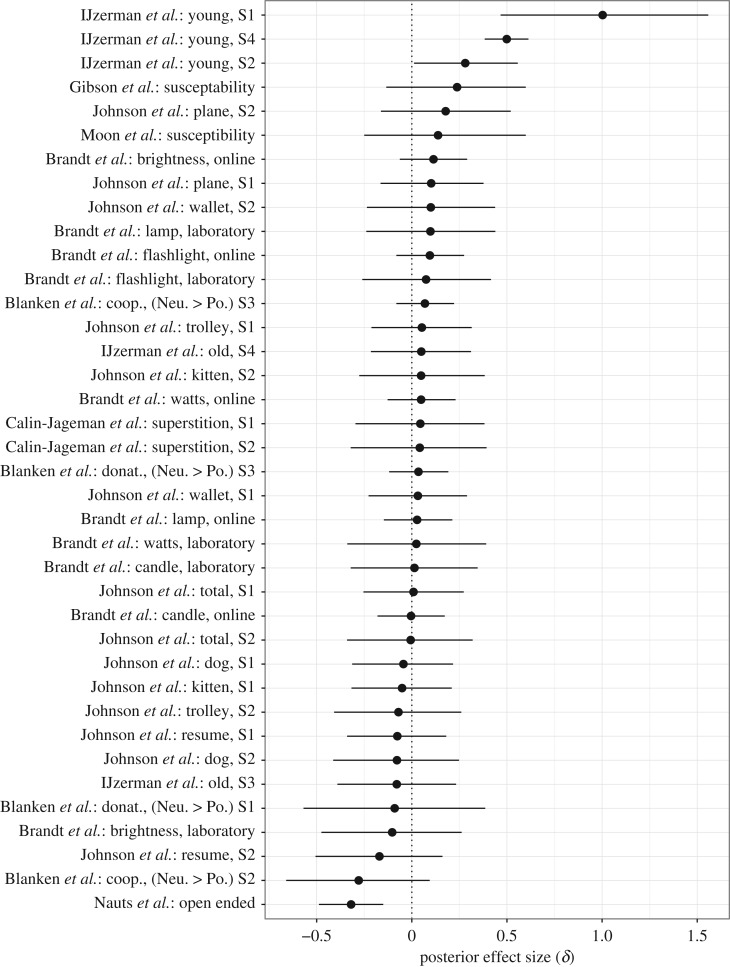
Individual Bayesian parameter estimation results for directed effect sizes *δ* for each of 38 experiments reported in the *Social Psychology* special issue that used *t*-tests. The posterior medians are indicated as dots and the central 95% credible intervals as vertical lines. The effect sizes were estimated using separate unrestricted models, but recoded such that they are predicted to be positive. Figure available at https://flic.kr/p/FQJrUr, under CC license https://creativecommons.org/licenses/by/2.0/.

**Figure 4.**
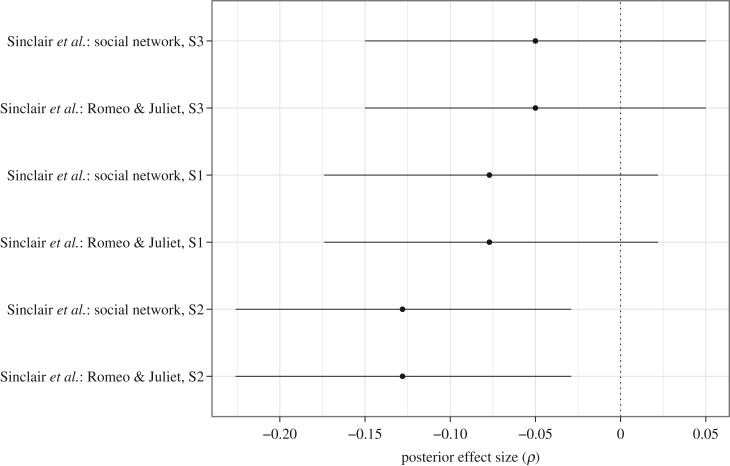
Individual Bayesian parameter estimation results for directed effect sizes *ρ* for each of six experiments reported in the *Social Psychology* special issue that used correlation tests. The posterior medians are indicated as dots and the central 95% credible intervals as vertical lines. The effect sizes were estimated using separate unrestricted models, but recoded such that they are predicted to be positive. Figure available at https://flic.kr/p/FqBRsm, under CC license https://creativecommons.org/licenses/by/2.0/.

The results for 44 directed effect sizes are shown in figures 3 and 4, with posterior medians indicated as dots and the central 95% credible intervals as horizontal lines. The posterior distributions in figures 3 and 4 were obtained from the unrestricted model (cf. figure 1*a*), recoded such that the prediction of interest stipulates the directed effect sizes to be positive. Furthermore, we have sorted the results in figures 3 and 4 according to the posterior median values, with the top-level entry showing the largest posterior median, and the bottom-level entry showing the smallest posterior median.

Figure 3 shows the results for the effect sizes *δ* separately for each of 38 replication studies and analyses using *t*-tests. From figure 3, we see that the estimated effect sizes are generally small, with only nine out of 38 studies yielding credible intervals that contained values |δ|≥0.5. Furthermore, we see that despite being designed for high power, many studies still yield credible intervals that are relatively wide—an indication that there remains considerable uncertainty about the true value of effect size under $H_{1}$ (cf. [55]). With small average effects and wide credible intervals, only three out of 38 central 95% credible intervals do not overlap with zero and fall in the intended direction. All three of these studies come from the IJzerman replication effort.

Figure 4 shows the results for the correlation effects *ρ* separately for each of six analyses reported by Sinclair *et al.* [56] using correlation tests. From figure 4, we again see relatively small effect size estimates and credible intervals that cover about 10% of the parameter range [−1,+1]. More importantly, only two credible intervals did not overlap with zero, both in the unintended direction.

As is evident from figures 3 and 4, only six out of the 44 credible intervals do not overlap with zero, and only three of these are in the predicted (positive) direction.

#### 3.1.2. Undirected effect sizes.

**Figure 5.**
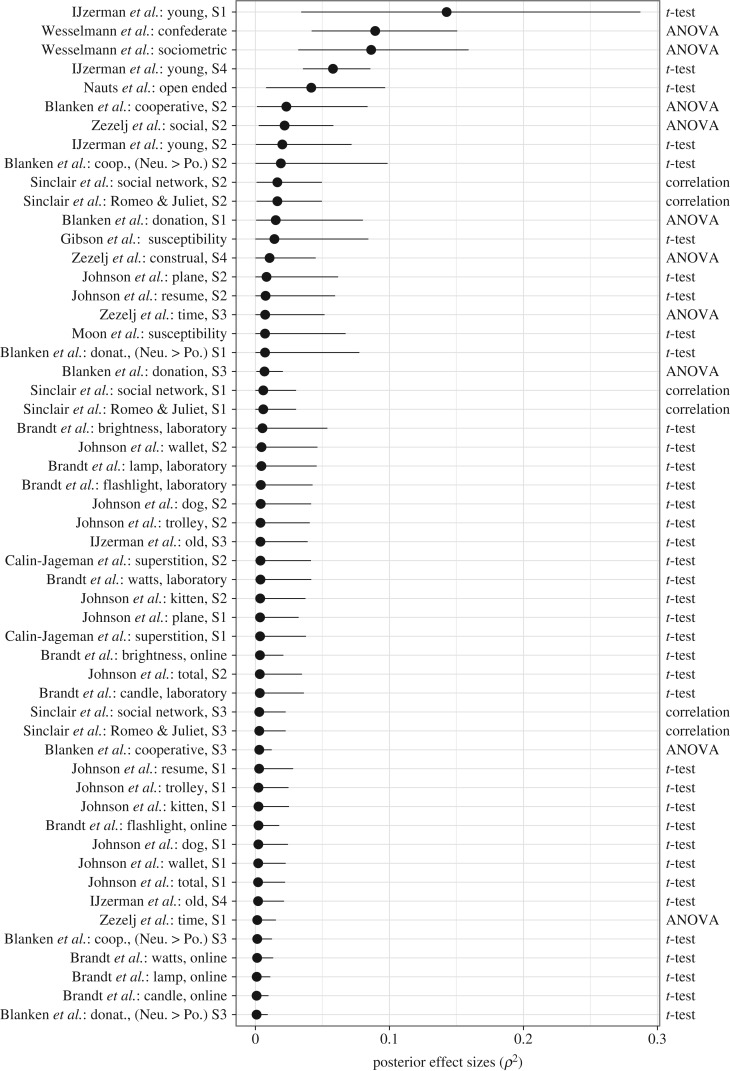
Individual Bayesian parameter estimation results for undirected effect sizes $\rho^{2}$ for each of 54 experiments reported in the *Social Psychology* special issue that used either *t*-tests, correlation tests or ANOVAs. The posterior medians are indicated as dots and the central 95% credible intervals as vertical lines. The effect sizes were estimated using an unrestricted model. Figure available at https://flic.kr/p/FJSqwH, under CC license https://creativecommons.org/licenses/by/2.0/.

**Figure 6.**
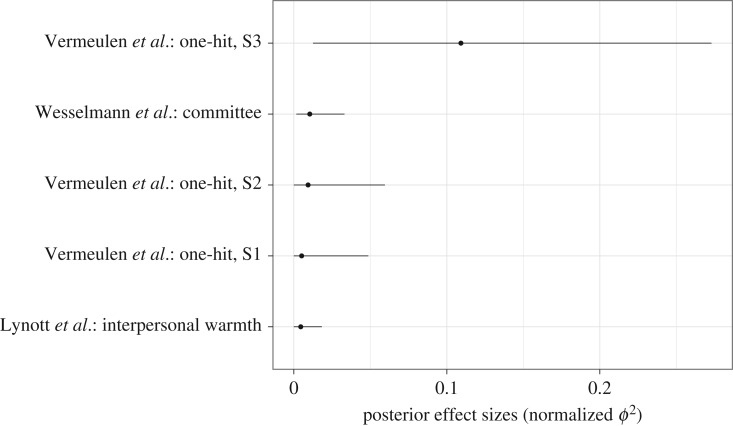
Individual Bayesian parameter estimation results for undirected effect sizes $\phi^{2}$ for each of five experiments reported in the *Social Psychology* special issue using contingency tables. The posterior medians are indicated as dots and the central 95% credible intervals as vertical lines. The effect sizes were estimated using an unrestricted model. Figure available at https://flic.kr/p/EVhMSS, under CC license https://creativecommons.org/licenses/by/2.0/.

The general pattern of results for the directed effect sizes is corroborated by the 59 undirected effect size estimates that are shown in figures 5 and 6, with posterior medians indicated as dots and the central 95% credible intervals as vertical lines. Figure 5 summarizes the results for the $\rho^{2}$ effect size estimates for the 54 studies that used either a *t*-test, a correlation test or an ANOVA, sorted according to the posterior median values. From figure 5, we see that only 17 out of the 54 credible intervals contained values larger than 0.05 (5% variance explained), and only three contained values larger than 0.10 (10% variance explained).^8^

Figure 6 shows the results for the $\phi^{2}$ effect size estimates for the five studies that used contingency tables, also sorted according to the posterior median values. We report squared *ϕ* values here since for a 2×2 contingency table Cramér's *ϕ* reduces to Pearson's $\rho_{\phi }$, making $\phi^{2}$ comparable to $\rho^{2}$ in this particular case.^9^

Figure 6 shows that only two credible intervals contained values larger than 0.05 (5% shared variance), and only one (i.e. the interval for the study by Vermeulen *et al.* [59]) contained values larger than 0.10 (10% shared variance). Note that the central 95% credible interval from the Vermeulen study is relatively wide, ranging from 0.01 to 0.27, indicating considerable uncertainty about the true value of this effect size.

### 3.2. Results from Bayesian parameter estimation: hierarchical analysis

**Figure 7.**
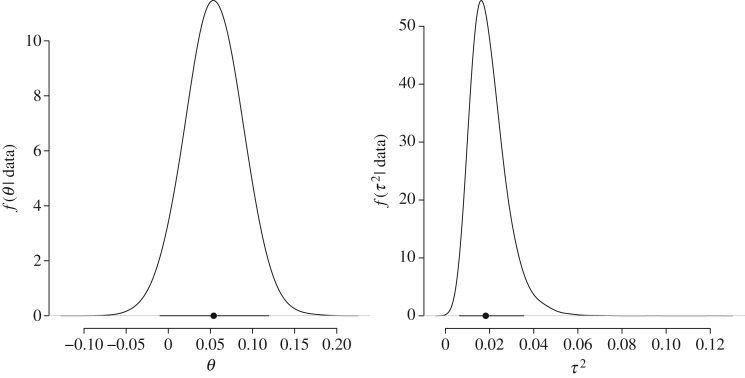
Posterior distributions for the group-level parameters of the hierarchical normal model describing the distribution of the 38 *t*-test effect sizes in our reanalysis. Posterior medians are indicated with dots and central 95% credible intervals are indicated with horizontal lines.

This section summarizes the results from the Bayesian random-effects meta-analysis of the effect sizes *δ* obtained from the 38 *t*-tests in the *Social Psychology* special issue. Figure 7 shows the posterior distributions for the two group-level parameters: the group mean effect *θ* and the between-study heterogeneity $\tau^{2}$. As is evident from figure 7, there is a relatively small overall group mean Cohen's effect size *θ*, with a posterior median of about 0.05 and a 95% central credible interval that overlaps with zero and ranges from −0.01 to 0.12. Furthermore, the between-study heterogeneity was relatively small; the posterior median for $\tau^{2}$ equals 0.019 and the central 95% credible interval equals [0.006,0.035].

**Figure 8.**
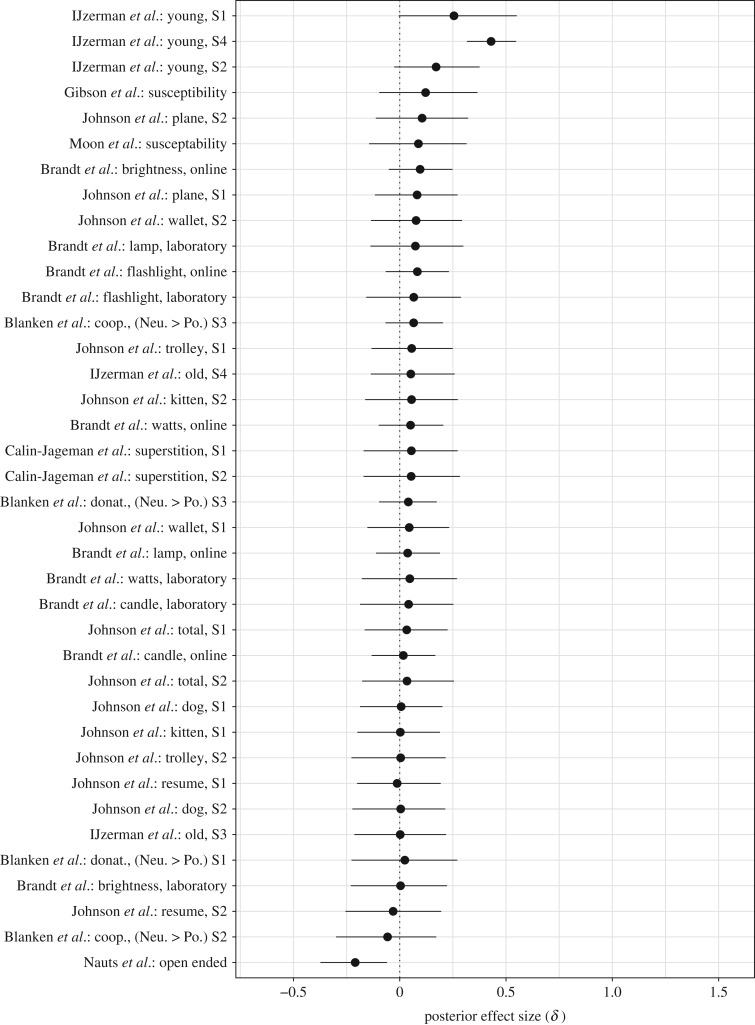
Individual results from a Bayesian random-effects analysis for the directed effect sizes *δ* from each of 38 experiments that used *t*-tests. The posterior medians are indicated as dots and the central 95% credible intervals as vertical lines. The effect sizes were estimated using a hierarchical model. Figure available at https://flic.kr/p/FqBRto, under CC license https://creativecommons.org/licenses/by/2.0/.

Figure 8 shows the hierarchically estimated credible intervals separately for each of the 38 studies, with posterior medians indicated as dots and the central 95% credible intervals as vertical lines. For a clear comparison, we have retained the same limits on the *x*-axis that were used to report the posterior distributions for individual experiments in figure 3, and sorted the results according to the posterior median values in figure 3. When we contrast the hierarchical estimates from figure 8 to the individual estimates from figure 3, we observe that the hierarchically estimated credible intervals are considerably shorter than the individual estimates. However, despite the substantial decrease in posterior uncertainty, only one of the hierarchically estimated credible intervals reported in figure 8 did not overlap with zero and fell in the expected direction. This regularity is the result of a substantial shrinkage effect that pulls individual posteriors towards the group mean *θ*. Shrinkage is particularly pronounced for effect size estimates that are relatively extreme and uncertain, as is exemplified by the first replication experiment reported in IJzerman *et al.* [33]. The individual estimate (shown as the top entry in figure 3) yields a posterior median of 1.0 and a central 95% credible interval of [0.467,1.556]; by contrast, the hierarchical estimate yields a much smaller posterior median of 0.26 and a central 95% credible interval of [−0.002,0.559].

### 3.3. Results from Bayesian hypothesis testing

**Figure 9.**
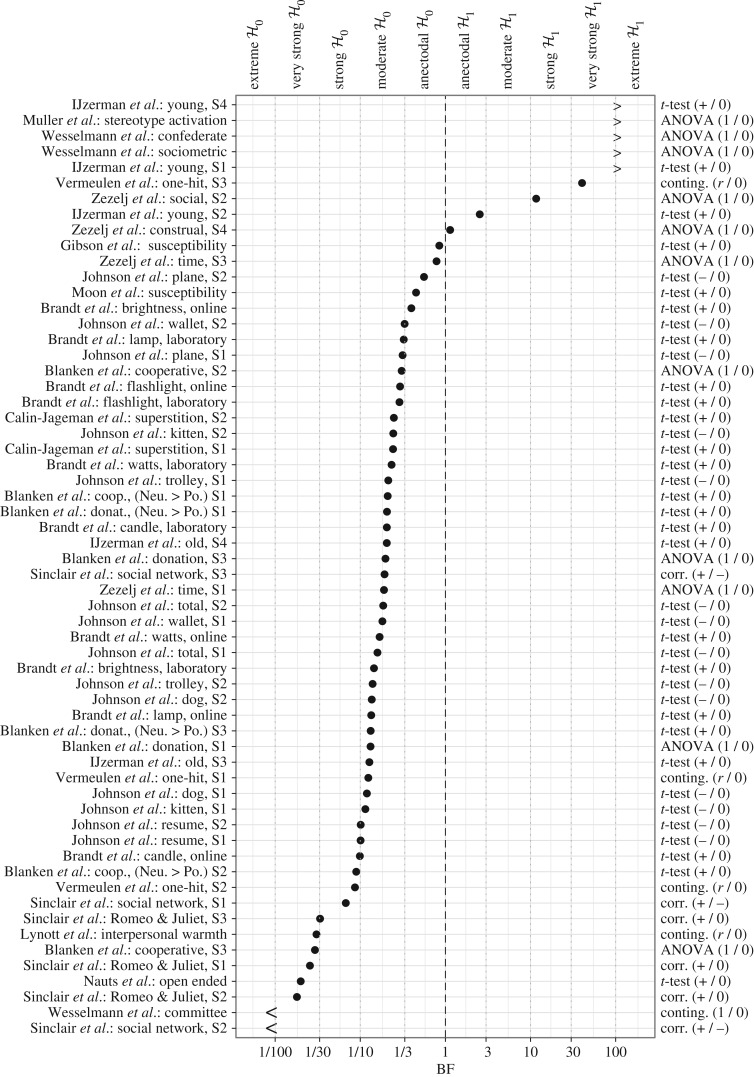
Default Bayes factors for 60 analyses reported in the *Social Psychology* special issue. The direction of the hypothesis is indicated in the right margin: ‘(1 / 0)’ refers to the two-sided ${\mathrm{BF}}_{10}$, ‘(+/0)’ refers to ${\mathrm{BF}}_{+0}$, ‘(−/0)’ refers to the one-sided ${\mathrm{BF}}_{-0}$, ‘(+/−)’ refers to the one-sided ${\mathrm{BF}}_{+-}$ and ‘(r/0)’ refers to ${\mathrm{BF}}_{r0}$ where $H_{r}$ refers to a predicted ordering of parameters. The top margin indicates the evidence categories proposed by Jeffreys [21]. Figure available at https://flic.kr/p/DZBTBj, under CC license https://creativecommons.org/licenses/by/2.0/.

This section reports the 60 default Bayes factors that test the directional hypothesis $H_{+}$ against the null hypothesis $H_{0}$. Figure 9 shows the Bayes factors sorted according to the degree to which they support $H_{+}$, with the top-level entry showing the most support for $H_{+}$ and the bottom-level entry showing the most support for $H_{0}$. The overall results are qualitatively consistent with those from the credible intervals: nine of the 60 Bayes factors showed evidence in favour of the alternative hypothesis, but only seven showed evidence for the alternative hypothesis that is more than anecdotal (i.e. ${\mathrm{BF}}_{+0}>3$). The remaining 51 Bayes factors showed evidence in favour of the null hypothesis. Out of these 51, a total of six indicated anecdotal support for $H_{0}$ (i.e. ${\mathrm{BF}}_{+0}\in (\frac{1}{3},1)$), 34 indicated moderate support for $H_{0}$ (i.e. ${\mathrm{BF}}_{+0}\in (\frac{1}{10},\frac{1}{3})$) and 11 indicate support for $H_{0}$ which is strong to extreme (i.e. ${\mathrm{BF}}_{+0}<(\frac{1}{10})$).

## 4. General discussion

We reanalysed the replication studies from the *Social Psychology* special issue on ‘Replications of Important Results in Social Psychology’. Our primary aim was to reanalyse and summarize the results from the individual contributions using Bayesian methods. We selected the *Social Psychology* special issue for several reasons. First, the data are publicly available on the Open Science Framework, greatly facilitating reanalysis. Second, the special issue has attracted much attention, but no Bayesian analysis or systematic overview of the results is currently available. Finally, the contributions to the special issue met several key desiderata: the findings under scrutiny were judged to be important (i.e. non-trivial, theoretically valuable); the experiments were designed taking into account feedback from the original authors; the data analysis plan was preregistered; and the studies were designed for high power. The set of replications from the *Social Psychology* special issue appears to represent an ideal scenario, one in which all prerequisites for successful replication have been met.

Our reanalysis featured three complementary Bayesian methods. *Individual-experiment parameter estimation* revealed that (i) for only three out of 44 directed effect sizes did the estimates go in the intended direction with central 95% credible intervals excluding zero and (ii) for only four out of 59 undirected effect sizes did the central 95% credible intervals contain values greater than 0.1 (10% variance explained). *Hierarchical random-effects meta-analysis* for 38 *t*-tests revealed that (i) the group-level mean effect size is near zero, and (ii) the hierarchically estimated credible intervals for the individual experiments showed a considerable shrinkage effect, underscoring the uncertainty surrounding the results obtained from individual experiments. Only one study yielded a central 95% credible interval that did not overlap with zero and fell in the intended direction. Finally, *Bayes factor hypothesis tests* revealed that for only seven out of 60 hypotheses did the Bayes factor indicate non-anecdotal evidence in favour of the hypothesis of interest.

### 4.1. Dangers of generalizing the results beyond the special issue

Across the empirical sciences there are recent signs of a ‘crisis of confidence’ [60] and a ‘crisis of reproducibility’ [61]. In psychology, several carefully conducted, large-scale replication initiatives have generally produced disappointing outcomes (e.g. [62]). In the light of these developments, it is tempting to view our results as another nail in the coffin for experimental psychology in general and social psychology specifically. However, such scepticism may be misplaced.

The strongest argument against blindly generalizing the present results to the entire field is that the studies from the special issue may not be representative. The special issue authors may have proposed the studies because they had prior knowledge—obtained from pilot experiments, colleagues or expert assessment of the plausibility of a given claim—suggesting the effect at hand may not replicate. This need not imply that the authors were intent on demonstrating a failure to replicate. Instead, the special issue authors may have been reluctant to propose a replication for well-established effects such as confirmation bias and social exclusion. Implicitly or explicitly, the authors may have felt that their time and effort would be spent more wisely on effects whose replication success was more uncertain.

Another argument against overgeneralizing the present results is that ‘No single replication provides the definitive word for or against the reality of an effect, just as no original study provides definitive evidence for it.’ [1, p. 139]. Indeed, when the replicability of specific findings is under scrutiny, a ‘many-labs’ approach is preferable (e.g. [54, 63–67]). Finally, one always has to keep in mind that ‘different results between original and replication research could mean that there are unknown moderators or boundary conditions that differentiate the two studies. As such, the replication can raise more questions than it answers.’ [1, p. 138]).

The sceptic might retort that the results from the special issue are consistent with those from the Reproducibility Project: Psychology [62] which featured a more random selection of studies in social psychology. Moreover, as the editors acknowledged, ‘This special issue contains several replications of textbook studies, sometimes with surprising results (Nauts, Langner, Huijsmans, Vonk, & Wigboldus, 2014; Sinclair, Hood, & Wright, 2014; Vermeulen, Batenburg, Beukeboom, & Smits, 2014; Wesselmann *et al.* 2014).’ [1, p. 139]. Indeed, figure 9 confirms that these studies cluster at the bottom, indicating that they provide evidence against the textbook effect. Lastly, even when the effects do not generalize to the field as a whole, the sceptic may argue that the general impression is still cause for concern.

In sum, we believe that it is premature, imprudent and unwarranted to generalize the pattern of results obtained from our Bayesian reanalysis for the studies from the *Social Psychology* special issue to all of social psychology, or even to a subdiscipline of social psychology. At the same time, it is also unwise to ignore the general message, which is that previously published results—even in psychology textbooks—may not replicate to the degree that one may hope. Cornerstone research demands careful replication, and the fact that previous research once ‘found’ the effect (e.g. N=20, p=0.04) is no reason to put blind faith in the result and consider it a proved fact of life (e.g. [68, fig. 4]).

### 4.2. Alternative statistical analyses

Our analysis efforts have demonstrated the ease with which default Bayesian analyses can be carried out using readily available software such as JASP, Stan and the BayesFactor package in R. In these default or ‘objective’ analyses, the prior distribution on effect size under $H_{1}$ is relatively broad and centred on zero (e.g. [20, 21, 69]).

An alternative ‘subjective’ Bayesian procedure is to use substantive knowledge and assign effect size under $H_{1}$ a more informative prior distribution. Compared with the default analyses, these subjective prior distributions are likely to be less spread out and are likely not to be centred on zero (e.g. [70]).^10^ The challenges and advantages of a subjective Bayesian analysis are beyond the scope of this article.

In general, there exist additional Bayesian methods to assess the degree of replication success (e.g. [47]). For instance, one may compare the effect size of the replication study to that of the original study [71, 72], or one may use the information about effect size from the original study to set up a prior distribution for the hypothesis test in the replication attempt (e.g. [47, 67]). We decided to report the results from the current set of methods because these methods are standard, fully developed for the tests of interest and easy to extend to research efforts that do not focus on replication.

Finally, it should be mentioned that the special issue included statistical methodology that is not yet available in its complete Bayesian form (e.g. [52]). In order to take full advantage of the Bayesian paradigm, it is imperative that Bayesian procedures are developed for the run-of-the-mill scenarios that confront researchers every day.

In closing, we believe that our Bayesian bird's eye view has provided an unambiguous overview of the results from the contributions to the *Social Psychology* special issue on ‘Replications of Important Results in Social Psychology’. This overview may motivate the field to take measures that ensure that published findings replicate at a higher rate than they do now (e.g. [73, 74]): a stronger focus on replication studies, more use of high-powered designs, standard adoption of preregistration and data sharing by default [75]. We also hope that future analyses of replication studies will be more inclusive by employing a range of different, complementary techniques. When different statistical procedures support the same inference, this can only serve to reinforce one's confidence in the robustness and validity of the results.

## Footnotes

1 Although the editors may not have had Bayesian procedures in mind when they used the word ‘evidence’, the common sense interpretation of evidence as something that causes a change in opinion is consistent with the Bayesian statistical paradigm.

2 See http://andrewgelman.com/2015/04/21/feather-bathroom-scale-kangaroo/. A similar sentiment was expressed by Edwards *et al.* [39, pp. 215–216]: ‘Convention asks, “Do these two programs differ at all in effectiveness?” Of course they do. Could any real difference in the programs fail to induce at least some slight difference in their effectiveness? Yet the difference in effectiveness may be negligible compared to the sensitivity of the experiment. In this way, the conventional question can be given meaning, and we shall often ask it without further explanation or apology.’

3 Andrew Gelman wishes to state that he hates Bayes factors. The reasons for his aversion are detailed in Gelman & Rubin [40] and Gelman *et al.* [41, ch. 6] and mainly concern the practice of assigning prior mass to a single point from a continuous distribution, and the resulting sensitivity to the prior distribution (see also [42]).

4 Our goal in this paper is comparative, and hence we use models that are similar to the models from the original paper, in this case, a normal error model for ordinal data.

5 The SumStats module conducts Bayesian inference for statistical scenarios that are uniquely defined by a small set of summary statistics (e.g. the binomial, *t*-tests, linear regression and correlation).

6 We are currently working to expand JASP to accommodate prior distributions that are not centred on zero, thereby facilitating a more complete subjective specification.

7 We were unable to perform a Bayesian equivalent of the primary classical analysis in Nauts *et al.* [52], which involved a signed-rank test. Even though there has been some pioneering work for a Bayesian equivalent of this test (see [53]), currently this does not involve a Bayes factor. We therefore focused our efforts on the analysis of the open-ended descriptions (i.e. the *t*-test reported on p. 159).

8 The primary effect of interest in Müller & Rothermund [57] could not be properly partialled out.

9 The Wesselmann *et al.* [58] study featured a 3×3 contingency table; all other studies reported in figure 6 featured a 2×2 contingency table.

10 Note that it is imprudent to change the default analysis by drastically lowering the width of the prior distribution while keeping it centred at zero; such a change makes $H_{1}$ similar to $H_{0}$, and all but guarantees that the data are not diagnostic.

## Data accessibility

Our analyses and data files are available at https://osf.io/bqwzd/.
