# Correction to ‘A Bayesian bird's eye view of ‘Replications of important results in social psychology’

**DOI:** 10.1098/rsos.170085

**Published:** 2017-03-01

**Authors:** Maarten Marsman, Felix D. Schönbrodt, Richard D. Morey, Yuling Yao, Andrew Gelman, Eric-Jan Wagenmakers

## 1. Introduction

This correction is to expand the reference list of our publication *A Bayesian bird's eye view of the special issue* ‘Replications of important results in social psychology’. In the original work, we reanalysed the data from 14 studies published in a special issue for *Social Psychology*. Even though we referred to each of these 14 studies in our figures, we failed to include all of them in the reference list. The references that were omitted from the original publication are listed [1–7].
